# Supplementary material for: Magnetospirillum magneticum as a Living Iron Chelator Induces TfR1 Upregulation and Decreases Cell Viability in Cancer Cells
Source: Int J Mol Sci. 2021 Jan 6;22(2):498. doi: 10.3390/ijms22020498 (PMC7825404; doi:10.3390/ijms22020498)
Supplement: Supplementary file 1 [file ijms-22-00498-s001.pdf]

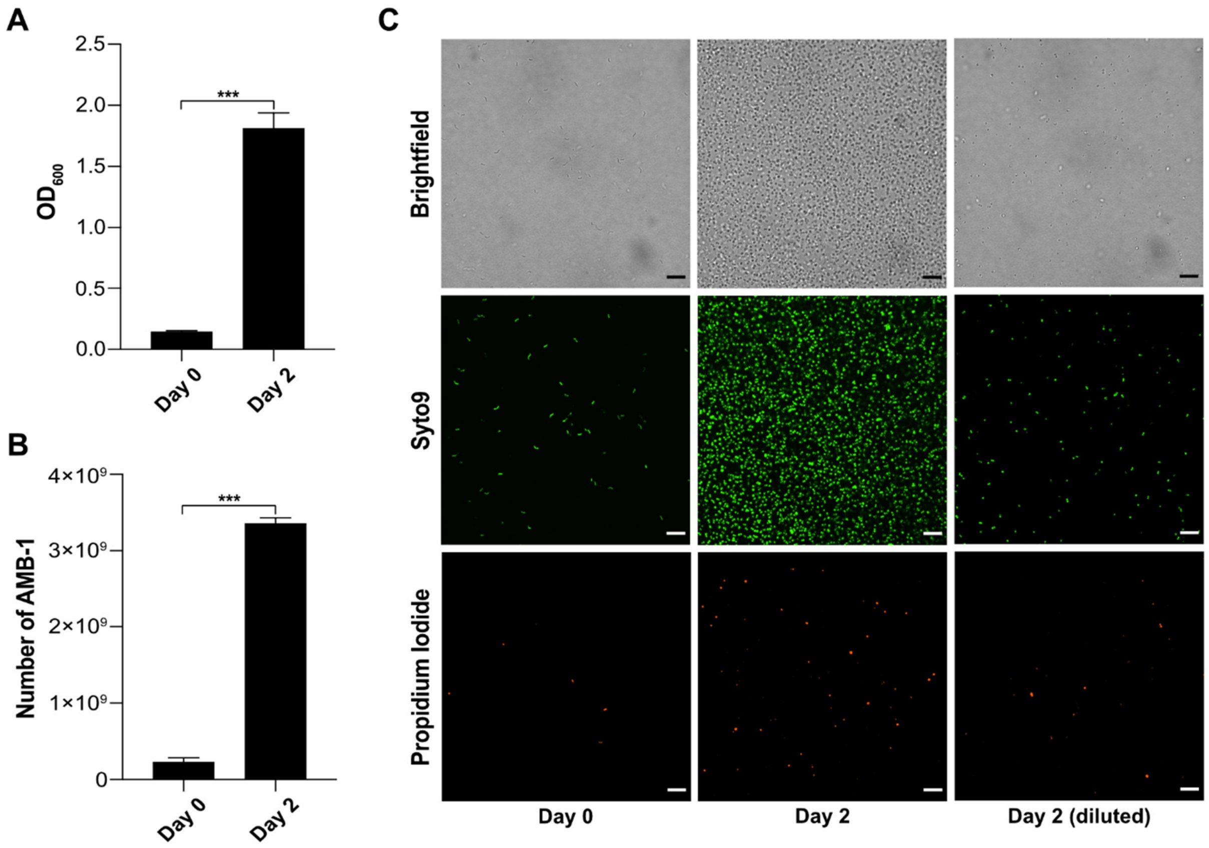

Figure S1: Quantification and imaging of proliferating AMB-1 bacteria at 37°C. **(A)** Optical density (OD_{600}) measurements of bacteria on Day 0 and Day 2 using a multimode microplate reader (n=6, individual replicates, *** p-value < 0.001) **(B)** Quantification of the increasing number of AMB-1 bacteria measured on Day 0 and Day 2, (n=3, technical replicates, *** p-value < 0.001). Counting of bacteria was performed using a Multisizer 4e (Coulter Counter). **(C)** Representative fluorescence images show AMB-1 bacteria stained with Syto9 (green) and Propidium Iodide (red). Bacteria were imaged at on Day 0 and Day 2, demonstrating the increase in viable cells. A sample diluted 1:15 of Day 2 was included to facilitate identification of single cells, (scale bar: 10 μm)

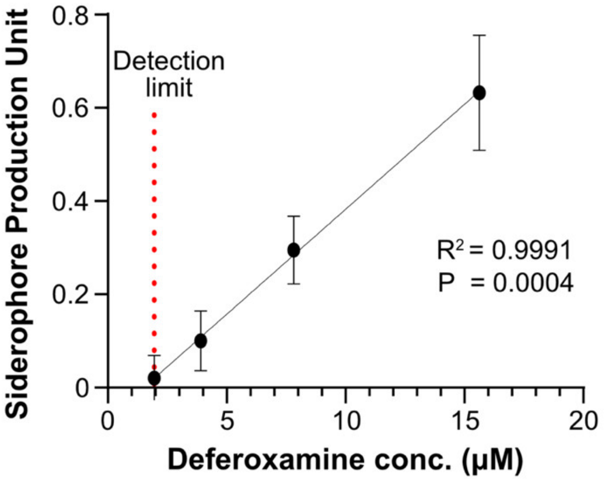

Figure S2: Calibration curve representing the siderophore production unit plotted against the concentration of deferoxamine (n= 3).

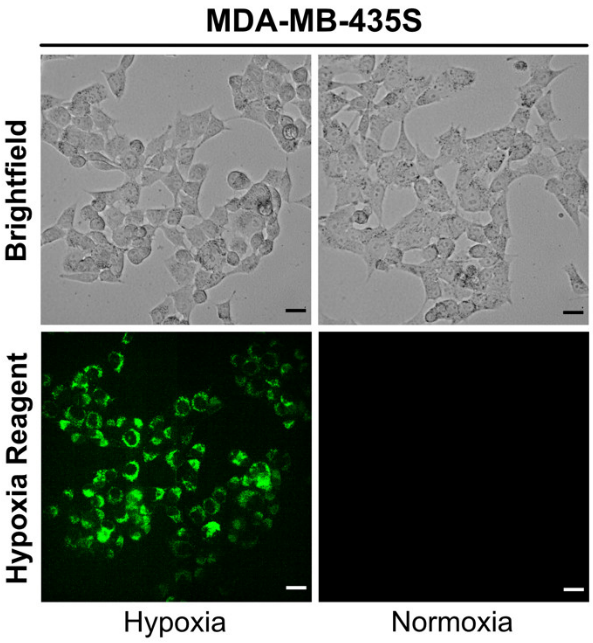

Figure S3: Comparison of in vitro cancer cell culture under either hypoxic or normoxic conditions. Representative fluorescence and brightfield images of MDA-MB-435S cells stained with Image-IT Green Hypoxia Reagent (green), (scale bar: 25 μm).

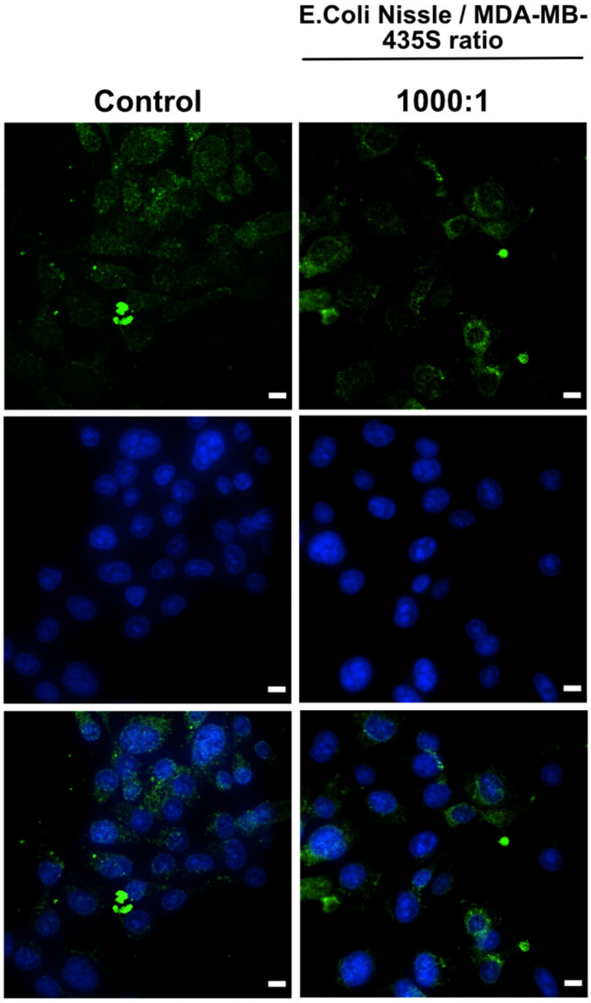

Figure S4: Immunofluorescence images of human melanoma cells co-cultured under hypoxic conditions for 48 hours with *E. coli* Nissle 1917. Images show MDA-MB-435S cells marked by anti-TfR1 antibody (green) and Hoechst 33342 (blue), (scale bar: 10 μm).

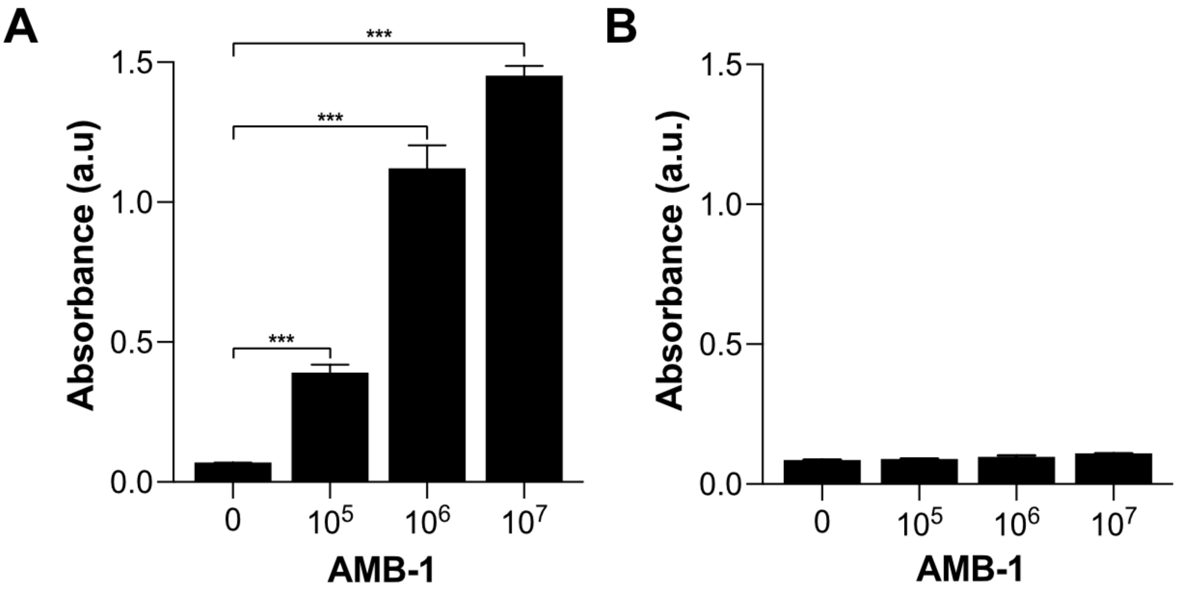

Figure S5: Determination of signal from MTB in wells following 0 and 3 washing steps. **(A)** Bacterial viability after 0 washing steps was determined using MTT assay and absorbance was measured at 540 nm. Signal is expressed as mean ± SD of 3 replicates. Ordinary one-way ANOVA test was used to assess statistical significance (*** p-value < 0.001). **(B)** Bacterial viability after 3 washing steps with DPBS was determined using an MTT assay and absorbance was measured at 540 nm. Signal is expressed as mean ± SD of 3 replicates.
